# Pedal Pull-Through Rail and Wingman-Assisted Coaxial Push for Entrapped Device Retrieval in Calcified Tibial Arteries

**DOI:** 10.1016/j.jaccas.2026.107255

**Published:** 2026-03-03

**Authors:** Akane Miyazaki, Masataka Yoshinaga, Keisuke Murata, Toru Araki, Kouki Matsuo, Takehiro Ito, Yoshihiro Sobue, Takashi Muramatsu, Wakaya Fujiwara, Eiichi Watanabe

**Affiliations:** a Department of Cardiology, Fujita Health University Bantane Hospital, Aichi, Japan; b Department of Cardiology, Fujita Health University Hospital, Aichi, Japan

**Keywords:** bailout technique, calcified lesion, complication, crosser, endovascular therapy, wingman

## Abstract

**Objective:**

To describe a bailout technique for retrieving an entrapped, fractured endovascular device fragment in severely calcified below-the-knee arteries when conventional snare retrieval is not feasible.

**Key Steps:**

1) Immediately stop manipulation and maintain the position of the antegrade guidewire holding the fragment (the “Golden Rule”); 2) obtain retrograde access; 3) advance a snare from the retrograde sheath to capture and externalize the existing antegrade guidewire, creating a pull-through rail without wire exchange; 4) advance a Wingman catheter antegradely over the rail; and 5) apply short, coaxial pushes to dislodge the fragment and retrieve it via the retrograde sheath.

**Potential Pitfalls:**

Retrograde distal puncture and sheath insertion are difficult without a sufficient vascular diameter in either the dorsal foot artery or posterior tibial artery. Avoid loss of the guidewire during procedure and vessel injury.

**Take-Home Message:**

Never surrender the workhorse wire holding the fragment; converting it into a pull-through rail enables safe coaxial dislodgement.

## Visual Summary

**Figure:**
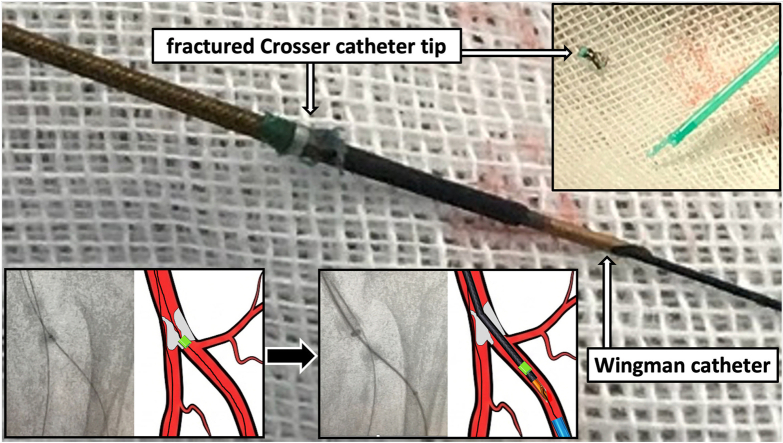

**Visual Summary:**
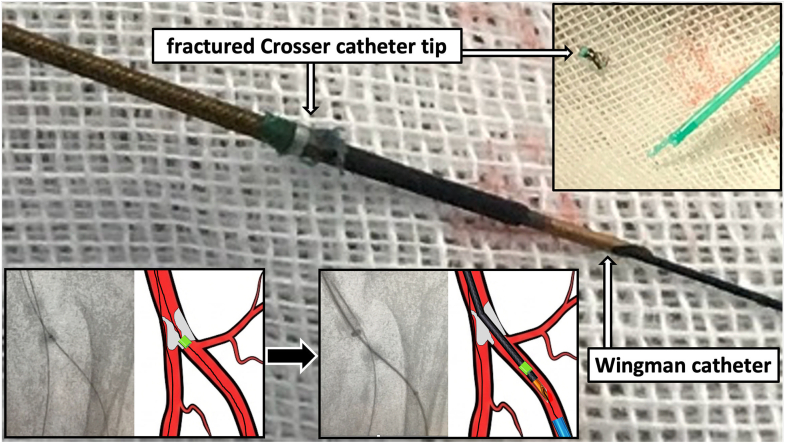
Schematic Illustration of the “Push-and-Release” Technique The fractured Crosser catheter tip retrieved on gauze. *Step-by-step mechanism:* Device fracture occurs on the antegrade wire. A retrograde snare captures the antegrade wire to create a pull-through rail. A Wingman catheter is advanced antegradely. The Wingman catheter applies a coaxial push to dislodge the fragment, which is then retrieved en bloc via the retrograde sheath.

Chronic limb-threatening ischemia in patients with diabetes and end-stage renal disease is frequently complicated by severe medial arterial calcification. These “rocky” below-the-knee lesions can be resistant to vessel preparation and can pose a high risk of device entrapment and fracture.^1^ Such complications are not unique to a single catheter type or model; rotational atherectomy burrs and balloon catheters have also become lodged in calcified tibial arteries.^2^, ^3^, ^4^, ^5^ The risk of entrapment rises sharply when operators encounter tiny, tortuous, or severely calcified vessels, as well as distal tapering, poor guidewire positioning, or mismatched burr-to-vessel size ratios.^3^ In peripheral arteries—particularly the tibial and tibioperoneal segments—these adverse features are commonplace, making device entrapment a plausible and under-recognized hazard. When a device becomes wedged in such a lesion, simple traction can force the hardware deeper into the calcified nodule.

**Take-Home Messages**

• In cases of device entrapment, never surrender the workhorse wire; it is the safest track for retrieval.
• Converting the entrapped wire into a pull-through rail via retrograde snaring allows for a controlled coaxial push technique to dislodge the fragment.

Historically, surgical extraction has been required in nearly half of the reported cases of atherectomy device entrapment,^3^ underscoring the need for reproducible endovascular bailout strategies. In this report, we describe a reproducible “How we did it” bailout technique using a pedal pull-through rail to retrieve a fractured Crosser catheter tip lodged in a severely calcified tibioperoneal trunk (TPT). Crucially, our method harnesses the existing antegrade guidewire to create the rail, adhering to the safety principle of never surrendering the wire on which the device is entrapped. We believe this strategy may help avoid escalation to open surgery and reduce the risk of distal embolization or vessel injury.

## Case Summary

**Figure 1:**
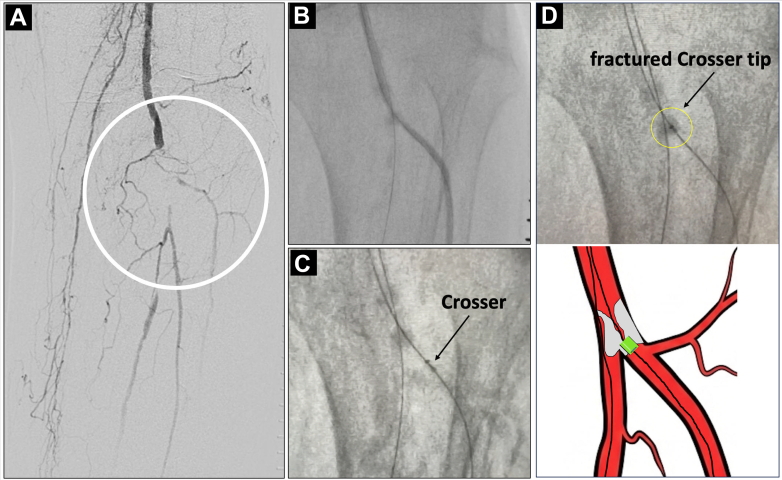
Fluoroscopic Images Showing the Fractured Crosser Catheter Tip (Arrows) Entrapped in the Calcified Tibioperoneal Trunk (A) Initial angiography showing a subtotal, heavily calcified lesion in the tibioperoneal trunk. (B) Persistent indentation (waist) despite high-pressure balloon angioplasty, indicating severe calcification. (C) The Crosser recanalization catheter became wedged in the calcified nodule. (D) The fractured Crosser tip was firmly entrapped on the guidewire at the tibioperoneal trunk bifurcation.

A 70-year-old woman on hemodialysis with a history of coronary artery bypass grafting presented with Rutherford class 4 chronic limb-threatening ischemia. One year prior, she had undergone endovascular therapy for a left superficial femoral artery (SFA) chronic total occlusion. She presented with acute limb ischemia (rest pain and pallor) during dialysis. Emergency angiography revealed SFA reocclusion and a subtotal, heavily calcified lesion in the TPT (Figure 1A). After surgical thrombectomy and SFA stenting, semicompliant balloon angioplasty was attempted; however, dilation was insufficient and the balloon indentation persisted at the calcified stenotic site (Figure 1B). We could not cross the TPT lesion with high-pressure or scoring balloons. A Crosser catheter (C.R. Bard)^6^ was used but became wedged in the calcified nodule during a maneuver to redirect it toward the anterior tibial artery (Figure 1C). The tip fractured and remained firmly entrapped on the guidewire (Figure 1D, Video 1).

## Procedural Steps

**Figure 2:**
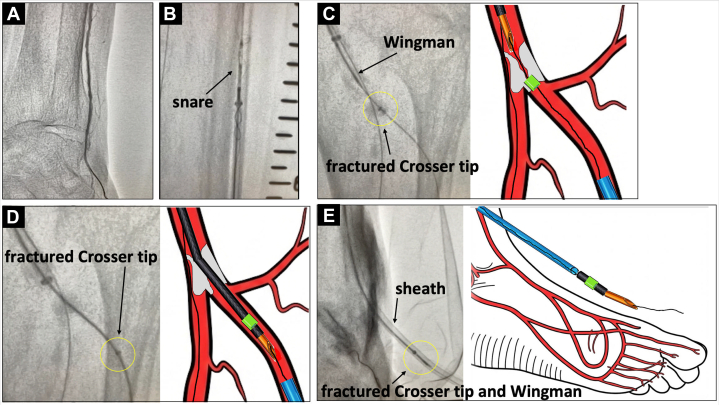
Pull-Through Rail Creation and Push-and-Release Maneuver (A) Ultrasound-guided retrograde dorsalis pedis access and sheath placement. (B) A snare advanced from the retrograde sheath captures and externalizes the antegrade guidewire, creating a through-and-through rail (Video 2). (C) A bevel-tipped Wingman catheter is advanced antegradely along the rail to the proximal edge of the entrapped fragment. (D) Short, coaxial pushes with the Wingman dislodge the fragment from the calcified shelf (Video 3). (E) The freed fragment is pushed into the retrograde sheath and removed en bloc (Video 4).
**Step 3: Create the Rail (Crucial Safety Maneuver).** A standard pull-through technique often involves passing a new retrograde wire. However, to avoid the risk of losing the track through the fragment, we used the existing antegrade wire. We advanced a snare from the retrograde pedal sheath up to the anterior tibial artery (Figure 2B). We successfully captured the tip of the antegrade guidewire (which was still holding the fragment) and externalized it through the pedal sheath. This created a stable through-and-through rail using the original workhorse wire, ensuring continuous control of the fragment without the risk of wire exchange (Video 2).
**Step 4: Coaxial “Push and Release.”** We advanced a bevel-tipped supportive Wingman catheter (ReFlow Medical) antegradely over the tensioned rail until it abutted the proximal end of the fragment (Figure 2C). Instead of pulling (which had caused the wedge effect), we applied short, coaxial pushes with the Wingman catheter. The rail provided the necessary support, and the push force successfully dislodged the fragment from the calcified shelf (Figure 2D, Video 3).
**Step 5: Retrograde Retrieval.** The dislodged fragment was pushed distally into the retrograde sheath by advancing the Wingman catheter (Figure 2E), then guided out of the body and retrieved as a single mass (Figure 3, Video 4). Final angiography demonstrated restored blood flow to the dorsal metatarsal and plantar arteries (Figure 4).

The bailout strategy is outlined below:

**Step 1: The “Golden Rule”—Freeze and Stabilize.** Upon recognizing the fracture, immediate cessation of manipulation is vital. We confirmed the fragment's location fluoroscopically. Most importantly, we decided not to withdraw the antegrade guidewire. This wire was the only lifeline tethering the fragment; removing it would turn a controlled entrapment into a free-floating foreign body retrieval with high embolization risk.
**Step 2: Establish Retrograde Pedal Access.** With the antegrade route blocked by the fragment and calcium, we obtained ultrasound-guided access to the dorsalis pedis artery. After confirming sufficient vessel diameter (>2.4 mm) via ultrasound, we inserted a 5-F high-capacity sheath (Parent Select 5082; Medikit). This sheath has a large inner diameter (2.12 mm; equivalent to a standard 6-F lumen) capable of accommodating the detached device fragment while also maintaining a low profile (outer diameter: 2.4 mm) (Figure 2A).

**Figure 3:**
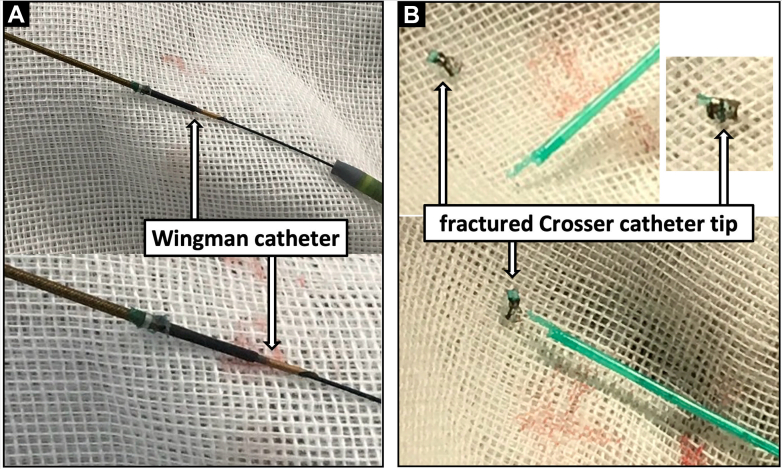
Photographs After Fragment Retrieval (A) The Wingman catheter (bevel-tipped support catheter) used to push the fragment. (B) The fractured Crosser catheter tip after successful retrieval, showing the detached distal metal segment (inset).

**Figure 4:**
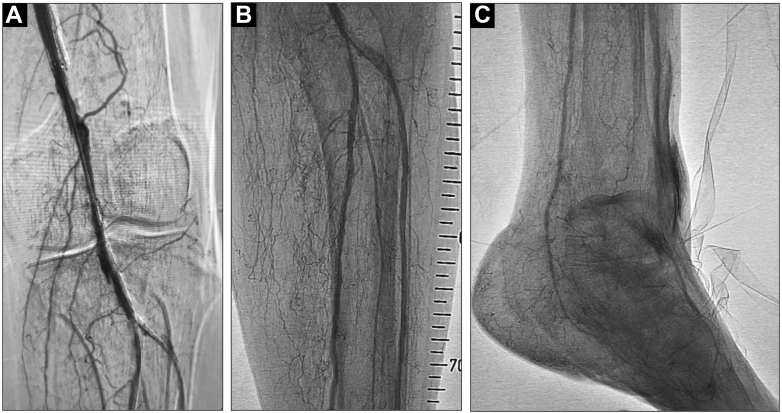
Final Angiography (A-C) Completion angiogram demonstrating restored flow in the dorsalis pedis and plantar arteries, with improved distal runoff.

## Discussion

Device entrapment in “rocky” calcified below-the-knee lesions is a nightmare scenario. Patients with end-stage renal disease frequently develop severe medial calcification of the tibial arteries, rendering lesions resistant to balloon angioplasty and increasing the risk of device entrapment. When the entrapped device cannot be crossed, snare retrieval becomes impossible. Retrograde access can facilitate retrieval by providing an alternative pathway. A recent case report described establishing retrograde tibial access and performing sequential balloon angioplasty around an entrapped atherectomy burr to liberate it.^3^ In the present case, the calcific napkin-ring stenosis prevented balloon passage; instead, we created a through-and-through rail and used a Wingman catheter. The Wingman catheter was selected for its unique bevel-tipped design and high pushability, which allowed it to act as a wedge between the calcified wall and the fragment. While other supportive catheters with sufficient lumen size—such as guide extension catheters or stiff microcatheters—could theoretically perform a coaxial push, the Wingman's specific tip geometry offers a mechanical advantage in dislodging wedged devices.

Our “How we did it” approach highlights 2 key technical principles:

1 **Secure the Workhorse Wire:** It is critical not to exchange the wire. If the wire holding the fragment is removed or lost, the fragment becomes a loose foreign body in a calcified vessel, making retrieval exponentially more difficult and dangerous.
2 **Retrograde Snaring of Antegrade Wire:** By snaring the antegrade wire from the pedal approach, a rail is created with zero risk of “losing” the fragment. This provides the extreme support needed for the “push-and-release” maneuver.

This rail transforms the unsupported catheter into a stable monorail, allowing transmission of significant axial force without catheter prolapse. The Wingman's beveled tip adds a mechanical advantage by acting as a wedge to pry the fragment from the calcified wall.^7^ Compared with the 2-wire snare technique,^4^ the pull-through rail does not require distal catheterization of the lesion, making it suitable for lesions that cannot be crossed. However, operators must balance wire tension carefully to avoid “cheese-wiring” through the arterial wall. Use of a polymer-jacketed wire and limiting tension to active pushing may mitigate this risk. Distal embolization is another concern; leaving the retrograde guide sheath across the fragment provides partial embolic protection.

Our experience suggests that the pedal pull-through rail and coaxial push technique can be a useful addition to the bailout algorithm for complex tibial interventions. Familiarity with alternative retrieval strategies may prevent escalation to open surgery when device entrapment occurs. If the fragment can not be dislodged using the coaxial push technique, alternative strategies include: 1) attempting to snare the proximal end of the fragment using a high-force microsnare if space permits; 2) deploying a balloon distal to the fragment to anchor and pull (though difficult in this uncrossable lesion); or 3) surgical extraction (arteriotomy), which is often required in up to 50% of entrapment cases in calcified vessels.^3^ Leaving the fragment in situ is considered high risk owing to the potential for migration and thrombosis.

## Potential Pitfalls

There are several pitfalls to consider:

• Retrograde distal puncture and sheath insertion are difficult without a sufficient vascular diameter in either the dorsal foot artery or posterior tibial artery.

*Solution*: Before performing complex endovascular treatment, it is essential to thoroughly assess the condition of the popliteal artery preoperatively.

• **Surrendering the Wire:** The most common error is attempting to withdraw the wire to “start over.” This often leaves the fragment stranded in the vessel.

*Solution*: Treat the entrapped wire as an immutable rail.

• **Wire Shearing:** Excessive sawing motion on a pull-through rail can cut through calcified tortuosity (“cheese-wiring”).

*Solution*: Maintain static tension only during the active “push” phase and use polymer-jacketed wires if possible.

• **Perforation:** Pushing a catheter against resistance carries perforation risk.

*Solution*: Ensure the rail is taut to straighten the vessel and apply force strictly coaxially.

## Conclusions

Retrieval of entrapped devices in calcified tibial arteries requires a strategy that prioritizes wire security. By snaring the existing antegrade wire from a retrograde approach, operators can establish a robust pull-through rail without risking wire loss. This rail facilitates a coaxial “push-and-release” maneuver, offering a safe and reproducible solution for complex entrapment scenarios.

## Funding Support and Author Disclosures

The authors have reported that they have no relationships relevant to the contents of this paper to disclose.

## Appendix

Video 1 Device Entrapment Fluoroscopic sequence showing the Crosser recanalization catheter becoming wedged in the calcified tibioperoneal trunk. The tip fractures and remains entrapped on the guidewire during attempted withdrawal.

Video 2 Creating the Pedal Pull-Through Rail Key maneuver: A snare is advanced from the retrograde pedal sheath. It captures the tip of the *existing* antegrade guidewire and externalizes it, creating a stable pull-through rail without losing control of the entrapped fragment.

Video 3 Coaxial “Push-and-Release” Maneuver A Wingman catheter is advanced antegradely over the tensioned pull-through rail. Short, coaxial pushes are applied to the proximal end of the fragment, successfully dislodging it from the calcified nodule.

Video 4 Retrograde Retrieval The dislodged fragment is pushed distally by the Wingman catheter, guided into the retrograde sheath, and removed from the body en bloc.
